# Supplementary material for: Effect of preconditioning on propofol-induced neurotoxicity during the developmental period
Source: PLoS One. 2022 Aug 19;17(8):e0273219. doi: 10.1371/journal.pone.0273219 (PMC9390907; doi:10.1371/journal.pone.0273219)
Supplement: Attachment — Submitted filename: Response to reviewers.docx [file pone.0273219.s005.docx]

**Response to Reviewers**

PONE-D-22-08060
**Effect of preconditioning on propofol-induced neurotoxicity during the developmental period**

PLOS ONE

Dear Dr. Shibuta

Thank you for submitting your manuscript to PLOS ONE. After careful consideration, we feel that it has merit but does not fully meet PLOS ONE’s publication criteria as it currently stands. Therefore, we invite you to submit a revised version of the manuscript that addresses the points raised during the review process.

Please submit your revised manuscript by Jul 30 2022 11:59PM. If you will need more time than this to complete your revisions, please reply to this message or contact the journal office at plosone@plos.org. When you're ready to submit your revision, log on to https://www.editorialmanager.com/pone/ and select the 'Submissions Needing Revision' folder to locate your manuscript file.

Please include the following items when submitting your revised manuscript:

A rebuttal letter that responds to each point raised by the academic editor and reviewer(s). You should upload this letter as a separate file labeled 'Response to Reviewers'.
A marked-up copy of your manuscript that highlights changes made to the original version. You should upload this as a separate file labeled 'Revised Manuscript with Track Changes'.

An unmarked version of your revised paper without tracked changes. You should upload this as a separate file labeled 'Manuscript'.

If you would like to make changes to your financial disclosure, please include your updated statement in your cover letter. Guidelines for resubmitting your figure files are available below the reviewer comments at the end of this letter.

If applicable, we recommend that you deposit your laboratory protocols in protocols.io to enhance the reproducibility of your results. Protocols.io assigns your protocol its own identifier (DOI) so that it can be cited independently in the future. For instructions see: https://journals.plos.org/plosone/s/submission-guidelines#loc- laboratory-protocols. Additionally, PLOS ONE offers an option for publishing peer-reviewed Lab Protocol articles, which describe protocols hosted on protocols.io. Read more information on sharing protocols at https://plos.org/protocols?utm_medium=editorial-email&utm_source=authorletters&utm_campaign=protocols.

We look forward to receiving your revised manuscript. Kind regards,

Dhermendra Tiwari Academic Editor PLOS ONE

Journal Requirements:
When submitting your revision, we need you to address these additional requirements.

1. Please ensure that your manuscript meets PLOS ONE's style requirements, including those for file naming. The PLOS ONE style templates can be found at

https://journals.plos.org/plosone/s/file?id=wjVg/PLOSOne_formatting_sample_main_body.pdf and

https://journals.plos.org/plosone/s/file?id=ba62/PLOSOne_formatting_sample_title_authors_affiliations.pdf

2. As part of your revision, please complete and submit a copy of the ARRIVE Guidelines checklist, a document that aims to improve experimental reporting and reproducibility of animal studies for purposes of post-publication data analysis and reproducibility: https://arriveguidelines.org/sites/arrive/files/Author%20Checklist%20- %20Full.pdf. Please include your completed checklist as a Supporting Information file. Note that if your paper is accepted for publication, this checklist will be published as part of your article.

**Response**

We submitted a copy of the ARRIVE guidelines with this letter. Thank you.

3. In your Data Availability statement, you have not specified where the minimal data set underlying the results described in your manuscript can be found. PLOS defines a study's minimal data set as the underlying data used to reach the conclusions drawn in the manuscript and any additional data required to replicate the reported study findings in their entirety. All PLOS journals require that the minimal data set be made fully available. For more information about our data policy, please see http://journals.plos.org/plosone/s/data-availability.

Upon re-submitting your revised manuscript, please upload your study’s minimal underlying data set as either Supporting Information files or to a stable, public repository and include the relevant URLs, DOIs, or accession numbers within your revised cover letter. For a list of acceptable repositories, please see http://journals.plos.org/ plosone/s/data-availability#loc-recommended-repositories. Any potentially identifying patient information must be fully anonymized.

**Response**

We submitted data set as files “PPF 100n.xlsx”, “PPF-PCーSR-PO.xlsx” and “PC-PPF(imaging)-PO.xlsx”

4. We note that you have included the phrase “data not shown” in your manuscript. Unfortunately, this does not meet our data sharing requirements. PLOS does not permit references to inaccessible data. We require that authors provide all relevant data within the paper, Supporting Information files, or in an acceptable, public repository. Please add a citation to support this phrase or upload the data that corresponds with these findings to a stable repository (such as Figshare or Dryad) and provide and URLs, DOIs, or accession numbers that may be used to access these data. Or, if the data are not a core part of the research being presented in your study, we ask that you remove the phrase that refers to these data.

**Response**

In accordance with your suggestion, we deleted “data not shown” and revised.

[Note: HTML markup is below. Please do not edit.] Reviewers' comments:
Reviewer's Responses to Questions
**Comments to the Author**

1. Is the manuscript technically sound, and do the data support the conclusions?

The manuscript must describe a technically sound piece of scientific research with data that supports the conclusions. Experiments must have been conducted rigorously, with appropriate controls, replication, and sample sizes. The conclusions must be drawn appropriately based on the data presented.

Reviewer #1: Partly Reviewer #2: Yes Reviewer #3: No

2. Has the statistical analysis been performed appropriately and rigorously?

Reviewer #1: Yes
Reviewer #2: Yes
Reviewer #3: Yes

3. Have the authors made all data underlying the findings in their manuscript fully available?

The PLOS Data policy requires authors to make all data underlying the findings described in their manuscript fully available without restriction, with rare exception (please refer to the Data Availability Statement in the manuscript PDF file). The data should be provided as part of the manuscript or its supporting information, or deposited to a public repository. For example, in addition to summary statistics, the data points behind means, medians and variance measures should be available. If there are restrictions on publicly sharing data—e.g. participant privacy or use of data from a third party—those must be specified.

Reviewer #1: Yes Reviewer #2: Yes Reviewer #3: Yes

4. Is the manuscript presented in an intelligible fashion and written in standard English?

PLOS ONE does not copyedit accepted manuscripts, so the language in submitted articles must be clear, correct, and unambiguous. Any typographical or grammatical errors should be corrected at revision, so please note any specific errors here.

Reviewer #1: Yes Reviewer #2: Yes Reviewer #3: Yes

5. Review Comments to the Author

Please use the space provided to explain your answers to the questions above. You may also include additional comments for the author, including concerns about dual publication, research ethics, or publication ethics. (Please upload your review as an attachment if it exceeds 20,000 characters)

Response to Reviewer #1:

Thank you very much for your valuable comments. Your comments have helped us improve our manuscript. We have stated the changes made to the manuscript and have answered your questions below. I hope these revisions meet your approval.

All abbreviations should be used full name in first using in abstract, introduction, and ... .

**Response**

We confirmed that all abbreviations were spelled out at their first occurrence, as per your comments.

Why was the PPF (PPF-PC) at 100 nM or 1 μM concentration diluted DMSO+PBS regimen chosen?

**Response**

PPF at 1 μM did not kill neurons significantly in our preliminary experiment. This concentration was the maximum concentration that did not affect the survival rate of the primary cultured neurons. However, 10 μM PPF killed neurons significantly. We also used PPF at 100 nM in case PPF 1μM affected neuronal survival (but it did not) or PPF-PC improved survival rate (but it did not).

Why were the 17-day chosen to remove the fetuses?

**Response**

Cultures of the embryonic and fetal mammalian central nervous system have been used in many experiments due to its reliability, while the *in vitro* maintenance of adult mammal neurons has hitherto been largely unsuccessful. We identified that E16–E17 was the best survival rate until 28DIV. The brains of fetuses on E<15 were too small to remove, and therefore, sometimes, we could not collect as many neurons as we anticipated.

What was the criteria for identifying a neuron?

**Response**

As we previously described, in order to confirm the purity of the neuronal culture, cells were immunostained with anti-MAP2 or anti-GFAP antibody, before and after the experiment. More than 97% expressed MAP2 and less than 2% of the cells expressed GFAP, regardless of the duration of the experiments. This demonstrated that most of the cells in our cultures were neurons. (J Neurosci Res. 72(5):613-21, 2003; Br. J. Anaesth. 104: 52-58, 2010)

The other techniques such as western blotting or RT-PCR should be used to confirm that cell death pathways (apoptosis, ..) is not involved in the administration of PPF.

**Response**

Moderate to high concentrations of PPF administration during the developmental period (till DIV8) are involved in neuronal death, as shown in our previous reports (Neurotoxicology 69: 320-9, 2018). In the present study, our main purpose was to investigate whether PC alleviates PPF-induced neurotoxicity but not to elucidate cell death pathways. To minimize the number of experimental animals we used, we did not investigate death pathways in the present study.

The magnification and quality of fig. 4 and Fig. 5 low. The scale bar or magnification should be added to the figures.

**Response**

We added scale bars in the Figures following your valuable comments.

What is the main difference between the present study and reference number 9?

**Response**

Reference 9 (our previous report, 2018) demonstrated that exposure to moderate – high doses of PPF during the early developmental period leads to neural death. Based on this finding, we tried preconditioning treatments to alleviate this PPF-induced neurotoxicity. PC with low-dose NMDA was effective against subsequent glutamate insults, as Sragovich et al (2012) demonstrated. However, our result showed that PPF-PC did not improve survival rate of the neurons from later moderate – high doses of PPF exposure during the early developmental period.

Thank you very much for your valuable comments, again.

Reviewer #2: Dear editor,
The study entitled “Effect of preconditioning on propofol-induced neurotoxicity during developmental period” presented a set of results showing that low concentrations of propofol using a preconditioning treatment did not alter the cytotoxic effects of propofol in moderate and high concentrations in fetal rat brain cells. The methodology used is satisfactory and in accordance with the conclusion presented. The manuscript is informative about the neurotoxicity shown by propofol during the embryogenesis of rats. After a minor review, the manuscript should be considered in PLOS ONE Journal.

Response to reviewer #2

Thank you for your valuable comments. Your comments encouraged us to improve our manuscript.

We have stated the changes made to the manuscript and have answered your questions below. I hope these revisions meet your approval.

Minor points:
In the introduction, please provide more robust data from literature where preconditioning treatments were positively functioning against a certain drug cytotoxicity.

**Response**

According to your helpful suggestion, we have added two references (Sragovich, S. et al 2012 and Navon et al 2012). The addition of these two references helped improve our manuscript significantly.

Please provide the chemical structure of propofol.

**Response**

According to your comment, we have provided the chemical structure of PPF as Figure 1.

Page 9, line 139: How many rat fetuses were used to obtain the 46 culture dishes of neurons?

Page 10, line 155: How many rat fetuses were used to obtain the 69 culture dishes of neurons?

**Response**

I am unable to provide the exact number of rat fetuses. However, we usually obtain approximately 20–30 cultured dishes from one pregnant rat. Each pregnant rat has 4–12 fetuses. In the present study, we confirmed that we purchased and used 12 pregnant rats in total. We mentioned this in the materials and method section of the revised manuscript.

Reviewer #3: The present study describes some interesting phrmacology of propofol in its neurotoxic capacity in the early development in experimental animals.
The works are interesting for further considerstion. However, befor the animal study, some basic and bridge translation between in vivo and in vitro are required. The [Ca2+] internalization via its specific chanels are not defined, although the event is extremely important. In addinio, its coupling with Na+ and K+ trsansporters are not explained through the experimental design. Other basic approaches they assessed are encouraged.

In conclusion, the study is interesting in our readers but the present form is too premature to justify the claims.

**Response**

Thank you very much for your valuable comments.

The involvement of L-type calcium channels in PPF-induced neurotoxicity has already been reported by Kahraman et al (2008). Our preliminary studies also showed the same result, therefore, we did not perform further investigations on this. We understand that a major limitation of this experiment is that the degree of effect on cortical neurons *in vitro* may not necessarily correlate with the neurotoxic effect *in vivo*. In addition, we comprehend the importance of the involvement of Na^+^ and K^+^ transporters in PPF-induced neurotoxicity to clarify the neurotoxic mechanism as you mentioned. We are greatly inspired by your valuable comments for our future study. Still, we believe the alleviation of neurotoxicity is one of the main interests of not only anesthesiologists, pediatricians, and obstetricians, but all medical doctors. Since preconditioning is considered to be one of the useful therapies, in addition to hypothermia and medication as a neuroprotection tool, we believe PlosOne will provide the appropriate audience for our research.

6. PLOS authors have the option to publish the peer review history of their article. If published, this will include your full peer review and any attached files.

If you choose “no”, your identity will remain anonymous but your review may still be made public.
**Do you want your identity to be public for this peer review?** For information about this choice, including

consent withdrawal, please see our Privacy Policy. Reviewer #1: No
Reviewer #2: **Yes:** Leonardo Pereira Franchi Reviewer #3: **Yes:** Cheorl-Ho Kim

[NOTE: If reviewer comments were submitted as an attachment file, they will be attached to this email and accessible via the submission site. Please log into your account, locate the manuscript record, and check for the action link "View Attachments". If this link does not appear, there are no attachment files.]

While revising your submission, please upload your figure files to the Preflight Analysis and Conversion Engine (PACE) digital diagnostic tool, https://pacev2.apexcovantage.com/. PACE helps ensure that figures meet PLOS requirements. To use PACE, you must first register as a user. Registration is free. Then, login and navigate to the UPLOAD tab, where you will find detailed instructions on how to use the tool. If you encounter any issues or have any questions when using PACE, please email PLOS at figures@plos.org. Please note that Supporting Information files do not need this step.

*In compliance with data protection regulations, you may request that we remove your personal registration details at any time. (Remove my information/details). Please contact the publication office if you have any questions.*
